# Bioluminescent Ratiometric Indicator for Analysis of Water Hardness in Household Water

**DOI:** 10.3390/s20113164

**Published:** 2020-06-02

**Authors:** Md Nadim Hossain, Ryuichi Ishida, Mitsuru Hattori, Tomoki Matsuda, Takeharu Nagai

**Affiliations:** 1 The Institute of Scientific and Industrial Research (SANKEN), Osaka University, 8-1 Mihogaoka, Ibaraki 567-0047, Japan; nd6@sanken.osaka-u.ac.jp (M.N.H.); i.doradoradora1@gmail.com (R.I.); 8tr@sanken.osaka-u.ac.jp (M.H.); tmatsuda@sanken.osaka-u.ac.jp (T.M.); 2 Department of Biotechnology, Graduate School of Engineering, Osaka University, 2-1 Yamadaoka, Suita 565-0871, Japan

**Keywords:** bioluminescence, indicator ratiometry, water hardness, softener, smartphone

## Abstract

Water hardness (WH) is a useful parameter for testing household water, such as drinking, cooking, and washing water. Many countries around the world use pipeline water in their houses, but there is a need to monitor the WH because hard water has a negative impact on appliances. Currently, WH is often measured using chemical dye-based WH indicators, and these techniques require expensive equipment, and trained personnel. Therefore, a low-cost and simple measurement method has been desired. Here, we report LOTUS-W, which consists of a luciferase, Nanoluc, a yellow fluorescent protein Venus, and a Ca^2+^/Mg^2+^ detection domain of human centrin 3. The binding of Ca^2+^/Mg^2+^ to this indicator changes the conformation of human centrin 3, and induces bioluminescence resonance energy transfer (BRET) from Nanoluc to Venus, which changes its emission spectrum about 140%. The dissociation constants of LOTUS-W for Ca^2+^/Mg^2+^ are approximately several mM, making it suitable for measuring WH in the household water. With this indicator in combination with a smartphone, we have demonstrated that it is possible to evaluate WH easily and quickly. This novel indicator has the potential to be used for measuring not only household water but also water used in the food industry, etc.

## 1. Introduction

Natural water contains several minerals, such as calcium, magnesium, potassium, phosphate, and carbonate. These minerals are generally added when natural water passes through soils and rocks containing large mineral deposits. Water with high concentrations of polyvalent cations (>+1), such as Ca^2+^ and Mg^2+^, is called hard water [1]. Other ions such as Al^3+^, Fe^2+^, Sr^2+^, Zn^2+^, and Mn^2+^ are also associated with water hardness (WH). However, their concentrations are much lower than Ca^2+^ and Mg^2+^. WH is determined based on the total concentration of Ca^2+^ and Mg^2+^ in parts per million (ppm) or milligrams per liter (mg/L) [2,3]. WH is classified into soft water (60 mg/L or less), moderately hard water (60–120 mg/L), hard water (120–180 mg/L), and very hard water (over 181 mg/L) by the World Health Organization (WHO) [1,4].

According to the United States Geological Survey (USGS), domestic water can be categorized into indoor and outdoor uses of household water. Generally, indoor household water is for drinking, food processing, bathing, flushing toilets, washing clothes and dishes, and outdoor uses are for watering lawns, gardens, and maintaining pools. Household water is provided by public suppliers or self-supplied. In the USA, 283 million people (nearly 99%) collect their household water from the public water supply system through the pipeline (tap), and 42.5 million people (1%) privately manage their domestic water from fresh groundwater sources [5]. The Environmental Protection Agency (EPA) is monitoring the quality of household water provided by the public supply system, but self-supplied owners are responsible to check their own water quality such as water hardness [6,7]. From a geographical viewpoint, the level of household water hardness is not the same throughout the country [8,9]. Many European and North American people prefer to drink commercially available bottled waters [1], but the maintenance of household waters is still important for the long life of appliances. For example, people who live in hard water areas are suffering from the solubility problem of surfactants (detergents) and soap, damaging effects on the household appliances such as the laundry machine, staining on the bathtub and toilet bowl, spots on dishes and glassware, and it can also cause dry skin [10,11,12,13]. To avoid these problems. water softener devices are used in many residential areas to remove the effects of high Ca^2+^ and Mg^2+^ from the hard water by replacing two Na^+^ [1]. This device contains ion exchange resins that bind the Ca^2+^/Mg^2+^. After softening a large quantity of hard water, the exchange medium accumulates a large amount of Ca^2+^ and Mg^2+^, and causes the internal degradation of this ion exchange resin that leads to ineffective Ca^2+^/Mg^2+^ removal in household water [14,15,16]. To detect such situations, it is important to routinely determine the WH when using water softening devices and household waters by a rapid and low-cost sensing system.

For the determination of household WH, chemicals such as eriochrome black t, pontachrome black TA, eriochrome-schwartz-T, ethylenediaminetetraacetic acid, and bismuth oxoiodide have been used [14,17,18]. These chemical compounds detect WH by changing color at titration. However, these methods are affected by other ions and are time-consuming [3]. Other techniques such as ion-selective electrodes, flame atomic absorption spectrometry, molecular absorption spectrophotometry, digital image calorimetry, digital image-based-flame emission, and ion-exchange chromatography have been used for the determination of hardness in water [19,20]. However, these techniques are required for specialized equipment and trained personnel [21].

Fluorescent protein-based indicators have been used as a tool for environmental applications such as metals detection in water and soil [22,23]. Förster resonance energy transfer (FRET) has been used for the development of indicators to monitor biological processes in real-time [24,25]. WH has also been determined by a chemical-based FRET indicator, where acriflavine (Acf) and rhodamine B (RhB) were used as a FRET donor and acceptor, respectively [20]. Although the indicator succeeded in the determination of WH, through the measurement of Ca^2+^ and Mg^2+^, another study from the same group also used this system to detect various salts such as KCl, NaCl, FeCl_3_, FeSO_4_, and AlCl_3_, suggesting that the indicator has low specificity for Ca^2+^ and Mg^2+^ [26]. In addition, the indicator has a narrow detectable WH range (0.03–0.2 mg/mL), making it difficult to investigate the wider range of WH in household waters. Furthermore, the fluorescent protein-based measurements require an excitation light with a specific wavelength as well as an interference filter to block the excess excitation light into the detector, which brings an intricacy in the measurement at home use. These limitations have been successfully overcome by employing a bioluminescence resonance energy transfer (BRET)-based indicator, where the luciferase generates luminescence in the absence of excitation light. Upon the binding of analytes and luciferin (substrate), the BRET-based bioluminescent indicator shows enough photons for detection [27,28]. Their emission light can be applied as portable detection [29,30]. The selectivity and sensitivity of BRET-based indicators are convincing for the detection of bile acid, cholesterol detection, lactate, and salivary cortisol from biological fluids out of the cells via a smartphone camera [31,32,33]. Therefore, a smartphone-based bioluminescence assay offers to develop a bioluminescent protein-based indicator for the determination of WH in household water.

Here, we report on the development and application of a bioluminescent protein-based ratiometric WH indicator for the assessment of hardness in household waters. The binding affinities to Ca^2+^ and Mg^2+^ are suitable for measuring WH in household waters. In addition, this bioluminescent indicator is able to rapidly detect WH by using a portable device such as a smartphone.

## 2. Materials and Methods

### 2.1. General Molecular Biology

DNA oligonucleotides used for cloning and construction of this indicator in this study were purchased from Hokkaido System Science Co. Ltd. (Hokkaido, Japan), and are listed in Table S1. KOD-Plus DNA polymerase (TOYOBO Life Science Department, Osaka, Japan) was used for PCR amplification. PCR products were purified by phenol-chloroform extraction, followed by ethanol purification. Restriction digestion was performed with endonucleases (Takara Bio, Shiga, Japan or New England Biolabs, Ipswich, MA, USA), following the manufacturer’s recommended protocol. The digested products were purified using agarose gel electrophoresis followed by gel extraction using QIAEX II gel extraction kits (QIAGEN, Hilden, Germany). The extracted DNA fragments were ligated using T4 ligase in Rapid Ligation Buffer (Promega, Madison, WI, USA). Small-scale plasmid DNA was harvested from 1.5 mL of LB liquid bacterial culture using alkaline lysis and purified by phenol-chloroform extraction and ethanol precipitation. Large-scale plasmid DNA was obtained from the alkaline lysis method using a bacterial pellet from 200 mL of LB liquid culture, followed by isopropanol precipitation, PEG-8000 precipitation, and two rounds of phenol-chloroform extraction. Sequences of the insert in plasmid DNA were confirmed by dye terminator cycle sequencing using the BigDye Terminator v1.1 Cycle Sequencing kit (ThermoFisher Scientific, San Jose, CA, USA). The bioluminescent substrate Coelenterazine h was purchased from Wako Chemicals (Osaka, Japan), and furimazine was purchased from Promega (Madison, WI, USA).

### 2.2. Construction of Bacterial Expression Vectors

Venus cDNA and the circularly permuted (cp) Venus series (cp50, 157, 173, 195, and 229 Venus) were cloned from BRAC [34]. These cDNAs were amplified by PCR using a sense primer with an attached *Bam*HI site and a reverse primer with a *Sph*I site. The cDNA of NanoLuc (Nluc) was amplified with a sense primer with an attached *Sac*I site and a reverse primer with *Eco*RI. cDNA of human centrin 3 (HsCen3) was amplified from the expression vector magFRET-1 (Addgene, #50742) [35], using a sense primer with an attached *Sph*I site and a reverse primer with *Sac*I site. To obtain Venus/cpVenus-HsCen3-Nluc, amplified PCR fragments were digested with the enzymes and inserted into the pRSET_B_ (Invitrogen, Carlsbad, CA, USA) vector between *Bam*HI and *Eco*RI sites by four-piece ligations. Construction of Nluc-HsCen3-Venus/cpVenus was performed by replacing each of the genes. Deletion of the C-terminus of the HsCen3 indicator was performed by inverse PCR with primers listed in Table S1.

### 2.3. Expression and Purification of Proteins

WH indicator was expressed in *Escherichia coli* strain JM109 (DE3), and cultured at 23 °C for 69 h in 200 mL LB bacterial growth medium supplemented with 100 µg/mL carbenicillin. Cultured cells were suspended in PBS buffer, and lysed using a French press (ThermoFisher Scientific). The lysate was centrifuged (8000 rpm at 4 °C for 20 min), and recombinant proteins were purified from the supernatant using Ni-NTA agarose affinity columns (QIAGEN), followed by buffer exchange with 20 mM HEPES (pH 7.4) with the desalting column, PD-10 (GE Healthcare, Buckinghamshire, UK). After lysis, all protein purification processes were conducted on ice to avoid protein degradation. Protein concentration was determined by both the alkaline-denaturation method [36,37] and the Bradford assay.

### 2.4. In Vitro Characterization of LOTUS-W

The emission spectra of the WH indicator was measured by adding 5 µM coelenterazine h to 50 nM or 4 nM of protein using a spectrophotometer (PMA-12, Hamamatsu Photonics, Shizuoka, Japan) or a micro-plate reader (SH-9000, Corona Electric, Ibaraki, Japan). The emission spectra were normalized at the iso-emission point (506 nm) and Mg^2+^ and Ca^2+^ titrations were performed in solutions containing 10 mM MOPS (pH 7.2) and 100 mM KCl at 25 °C. After a full scan of the light emission spectrum from 400–650 nm, the ratiometric value (ratio) was obtained by dividing the acceptor (cpVenus) emission peak (525 nm) by the donor (Nluc) emission peak (455 nm). The dynamic range of the WH indicator was determined based on the ratio in the presence and absence of Ca^2+^/Mg^2+^. The dissociation constants (*K*_d_) for Mg^2+^ and Ca^2+^ were calculated by non-linear regression analysis of the ratio-based titration curve. Ratios were normalized with a maximum count. Sigmoidal-binding curves were fitted to the data using the Origin7 software (OriginLab Corporation, Northampton, MA, USA) to calculate using the Hill equation.

### 2.5. pH Measurement of LOTUS-W

To measure pH dependency, buffers with different pH values were prepared using 30 mM Na_2_B_4_O_7_·10H_2_O, and 30 mM C_6_H_5_Na_3_O_7_·2H_2_O [38]. The pH was adjusted in the range of 3.5–11.0 by the addition of HCl. Bioluminescence spectra were recorded using a microplate reader (SH-9000) at a final protein concentration of 10 nM.

### 2.6. Metal Ion Selectivity and Sensitivity of LOTUS-W

Purified LOTUS-W was prepared in buffers containing different metal ions with and without Mg^2+^ and Ca^2+^. Li^+^, K^+^, Mn^2+^, Fe^2+^, Co^2+^, Ni^2+^, Cu^2+^, Zn^2+^, As^3+^, Cd^2+^, and Pb^2+^ buffers were prepared by using LiCl, KCl, MnCl_2_, FeCl_2_, CoCl_2_, NiCl_2_, CuCl_2_, ZnCl_2_, AsCl_3_, CdCl_2_, and PbCl_2_, respectively, containing 10 mM MOPS (pH 7.2) and 100 mM KCl. Buffers with the same base and no additional metal ions were used as controls. Specificity was determined with 50 nM LOTUS-W by adding 10 mM Mg^2+^ or 10 mM Ca^2+^ in the presence of 3 µM (mostly harmful ranges) of metal ions. All assays were performed by using a microplate reader.

### 2.7. Temperature Calibration of LOTUS-W

The temperature sensitivity of LOTUS-W was analyzed using a spectrophotometer. The temperature of the sample was regulated by a temperature-sensitive electrode. Bioluminescence of a mixture containing 50 nM LOTUS-W and 5 µM Coelenterazine h, at temperatures ranging from 25–45 °C, was measured.

### 2.8. WH Determination by a Smartphone Camera

The WH scale was defined according to the guidelines of USGS/WHO (Table S2). Non-carbonate-based WH was calculated based on Ca^2+^ and Mg^2+^ concentrations (mg/L). Previous studies have calculated water hardness using certain equations [39,40]. For WH measurements, we prepared solutions containing 10 mM MOPS (pH 7.2) and 100 mM KCl. The equation for the calculation of the WH is described in the next section. WH of commercially available mineral waters, which were used for the measurements, is listed in Table S3 As a blank control, a solution with 10 mM MOPS (pH 7.2) and 100 mM KCl, without additional metal ions was used.

The bioluminescence reaction was performed in 96-well microplates with 50 µL of 4 nM LOTUS-W, 50 µL of WH buffer, and 100 µL of 5 µM coelenterazine h. The Galaxy S8+ (1220 × 10^4^ pixels, ISO: 800, and the F-score: 1.7, outer camera) smartphone was set at an exposure time of 15 ms. Images were taken in a dark box. The color balance (white balance) was set manually between 5000K and 5500K. The original RGB images were analyzed using MetaMorph (Molecular Devices, Sunnyvale, CA, USA), and Image J. The ratio was calculated from the averaged green and blue intensities, as shown previously [33]. All experiments were performed at 25 °C.

### 2.9. Calculation of Water Hardness

The total WH expressed as the concentration of calcium and magnesium ions are equal to the concentration of CaCO_3_. We calculated WH using a standard equation prescribed by WHO/EPS [41] and the Ministry of Health, Labor, and Welfare, Japan. (https://www.mext.go.jp/component/english/__icsFiles/afieldfile/2017/05/08/1385123_Minerals_in_tap_water_1.pdf)

Total hardness (TH) = hardness of calcium + hardness of magnesium

TH = [Ca^2+^] × (conversion rate of Ca^2+^ to CaCO_3_) + [Mg^2+^] × (conversion rate of Mg^2+^ to CaCO_3_)

TH = [mg/L of Ca^2+^] × 2.5 + [mg/L of Mg^2+^] × 4.1

Here, the molar masses of CaCO_3_, Ca^2+^, and Mg^2+^ are 100.1 g/mol, 40.1 g/mol, and 24.3 g/mol, respectively.

## 3. Results and Discussion

### 3.1. Development of the Ratiometric Bioluminescent LOTUS-W Indicator

**Figure 1:**
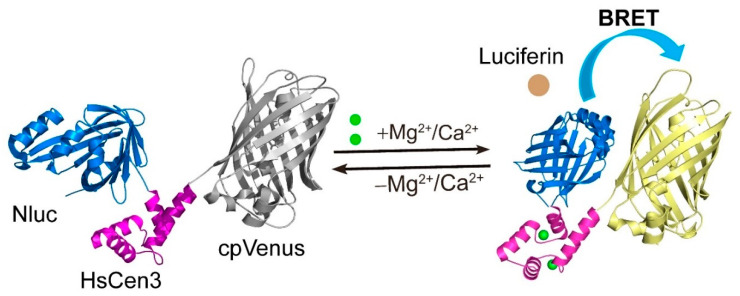
Schematic of Mg^2+^ and Ca^2+^ sensing mechanism in the water hardness indicator. HsCen3 (magenta) is an Mg^2+^ and Ca^2+^ binding domain, which is partially truncated for sensor function. HsCen3 is sandwiched between Nluc (blue), and cpVenus (gray) (left). Conformational changes in HsCen3, by binding of Mg^2+^ or Ca^2+^, induce spatial relocation of Nluc and cpVenus, increasing the efficiency of resonance energy transfer (cpVenus showing yellow).

Previously, we developed a genetically encoded bioluminescent indicator for monitoring the voltage of a cell membrane called Luminescent Optical Tool for universal sensing of voltage (LOTUS-V) [28]. This indicator detects the voltage through the voltage-sensing domain derived from voltage-sensing phosphatase, resulting in the variation of BRET between the bioluminescent protein, NanoLuc (Nluc), and the yellow fluorescent protein, Venus. Based on this indicator, we designed a novel WH indicator using Nluc, Venus, and a Ca^2+^/Mg^2+^ sensing domain human centrin 3 (HsCen3) [35] (Figure 1).

The Mg^2+^/Ca^2+^ binding domain HsCen3, which links between Cerulean and Citrine in the previously reported FRET-based indicator MagFRET, was used for the development of the novel indicator [35]. Following a trial in the development of the existing Ca^2+^ indicator, BRAC [34], twelve candidate WH indicators were constructed by replacing the Venus with circularly permuted Venus (cpVenus) variants (cp50Venus, cp157Venus, cp173Venus, cp195Venus, and cp229Venus) [42] with a deletion of ten amino acids from the C-terminus (Figure S1A, and Table S4). The binding of Ca^2+^/Mg^2+^ to this indicator changes the structure of human centrin 3, and induces bioluminescence resonance energy transfer (BRET) from Nluc to cpVenus. These candidates were purified and their luciferase activity was characterized by the addition of Mg^2+^. Based on the screening by dynamic range, we selected cp229Venus∆C10–HsCen3–Nluc (cpV229–HsCen3-NL), because it showed the highest dynamic range of about 28% (Figure S1B).

**Figure 2:**
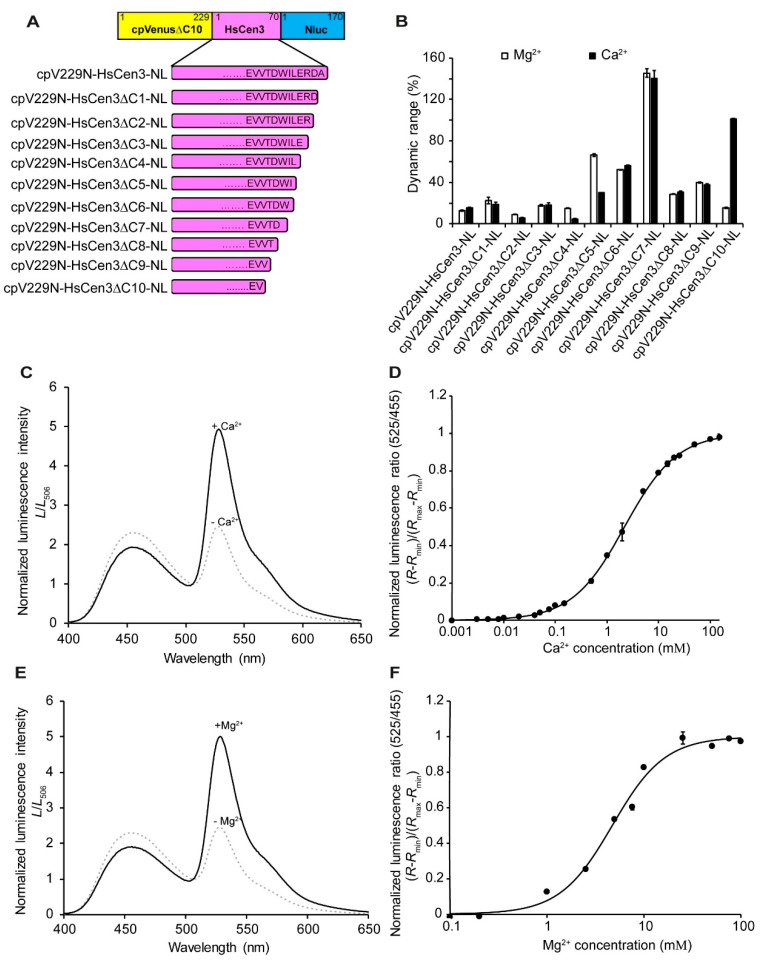
Development of ratiometric water hardness indicator and their in vitro properties (**A**) Domain structures of water hardness indicator candidates. HsCen3 with several deletions from the C-terminus was inserted between cpVenusΔC10 and Nluc. (**B**) Dynamic ranges of the variants were calculated from the emission ratio (525 nm/455 nm) with and without 10 mM Ca^2+^/Mg^2+^. cpV229N–HsCen3∆C7-NL showed the highest dynamic range. (**C**,**E**) Bioluminescence emission spectra of cpV229N–HsCen3∆C7-NL in the presence (black line) and absence (dashed line) of Ca^2+^ or Mg^2+^. (**D**,**F**) Titration curves of the emission ratio of cpV229N–HsCen3∆C7-NL. Bioluminescence emission ratios (525 nm/455 nm) with different concentrations of Ca^2+^ and Mg^2+^ have been plotted. The *K*_d_ of each ion was calculated from the emission ratios (525 nm/455 nm). Ratios were normalized by using the maximum ratio (*n* = 3).

To further improve cpV229–HsCen3-NL, 10 additional variants were created by truncation of HsCen3 at the C-terminus (Figure 2A), and their bioluminescent properties were analyzed from the signal changes in the presence and absence of Ca^2+^ and Mg^2+^ (Figure 2B and Figure S2). From the spectrum, cpV229–HsCen3∆C7-NL showed the maximum dynamic range of 140% and 145% (Figure 2C,E), respectively. Titration curves of the emission ratio indicated that the dissociation constant (*K*_d_) for Ca^2+^ was 2.1 mM (Hill coefficient, 0.85) and that for Mg^2+^ it was 4.7 mM (Hill coefficient, 1.6) (Figure 2D,F). The Mg^2+^ and Ca^2+^ affinity of LOTUS-W was similar to the MagFRET-5 indicator (*K*_d_ = 7.4 mM to Mg^2+^, and 1.6 mM to Ca^2+^) [35]. The dynamic ranges were higher (2 to 3 folds) than MagFRET-5.

### 3.2. Characterization of LOTUS-W Against Environmental Changes

**Figure 3:**
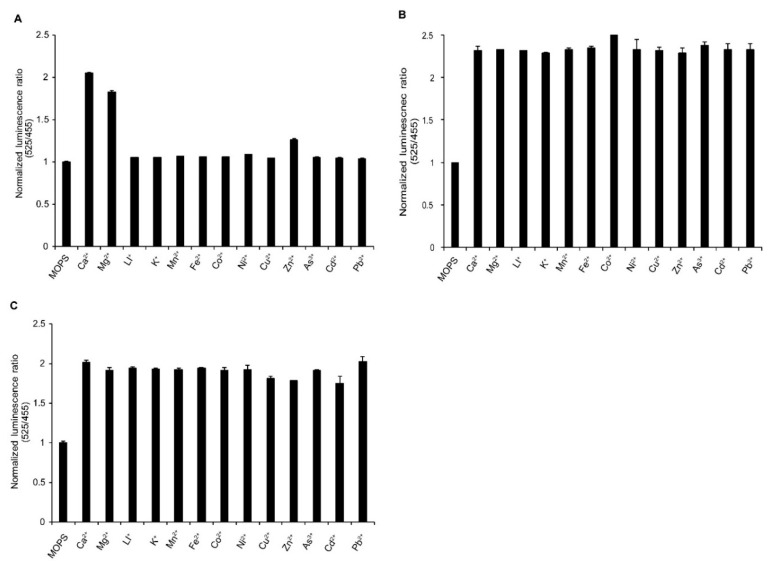
Specificity and selectivity of LOTUS-W for calcium and magnesium ions. (**A**) The specificity of LOTUS-W for Ca^2+^ and Mg^2+^. Emission ratios were measured in the presence of each metal ion. (**B**,**C**) The selectivity of LOTUS-W to Ca^2+^ (**B**) or Mg^2+^ (**C**), upon mixing with different metal ions. LOTUS-W was premixed with each metal ion, and Ca^2+^ or Mg^2+^ was added. Bioluminescence emission ratio was normalized (*n* = 3) to the count without additional metal ions (MOPS).

We confirmed the specificity of cpV229N_HsCen3∆C7-NL to Ca^2+^ and Mg^2+^ for other metal ions: Li^+^, K^+^, Mn^2+^, Fe^2+^, Co^2+^, Ni^2+^, Cu^2+^, Zn^2+^, As^3+^, Cd^2+^, and Pb^2+^. Comparing the metal ions, Ca^2+^ and Mg^2+^ induced noticeable changes in the bioluminescence ratio of cpV229N_HsCen3∆C7-NL (Figure 3A). Their selectivity did not change even when mixed with other metal ions (Figure 3B,C). Thus, cpV229N_HsCen3∆C7-NL showed high selectivity for Ca^2+^ and Mg^2+^.

Next, the pH-dependence of cpV229N_HsCen3∆C7-NL was analyzed between pH 3–11 (Figure S3). As the reaction to Ca^2+^ and Mg^2+^ did not fluctuate at different pH values, cpV229N_HsCen3∆C7-NL could be used for WH measurements over a wide pH range. The indicator is hypothesized to be useful in different temperature conditions. For this purpose, we also studied temperature dependency (Figure S4). The results suggested that cpV229N_HsCen3∆C7-NL stably worked in the range of 20–45 °C. Purified proteins are relatively fragile due to environmental factors such as heat and oxidation. Therefore, it is important to demonstrate the protein stability of LOTUS-W. We have performed time-course measurements with different incubations. This result supported us to prove that the LOTUS-W is stable in WH measurement at least one hour at room temperature (Figure S5). Thus, cpV229N_HsCen3∆C7-NL can measure WH under various environmental conditions. Based on these properties, we decided to use cpV229N_HsCen3∆C7-NL as a WH indicator by designating it as LOTUS-W.

### 3.3. WH Measurement by a Mobile Device

**Figure 4:**
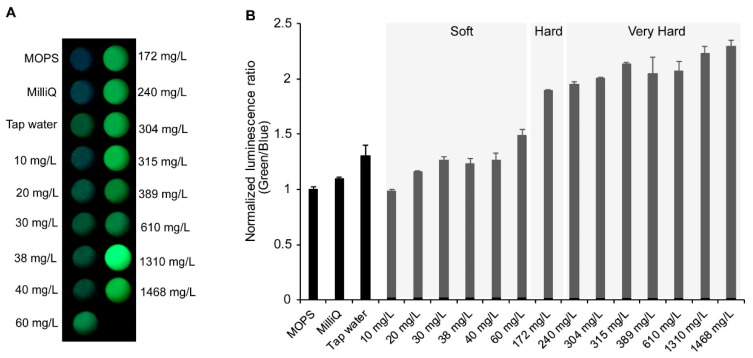
Detection of WH-dependent bioluminescence color change using a smartphone camera. LOTUS-W and water samples were reacted on a 96-well plate with a substrate, and images were taken using a smartphone camera. The image from each sample area (**A**) was separated into red-green-blue channels, and the intensity ratio of green to blue was calculated (**B**). From the results of the commercially available drinking water with different WH, the scale of WH of the tap water was assessed. Milli-Q water was used as the control. Each bioluminescence emission ratio was normalized to the count without additional water (MOPS) (*n* = 3). The classifications of WH are indicated in gray boxes.

Recently, imaging based on mobile devices has been offered as an attractive analytical technique in biological and environmental monitoring [43]. Especially, the smartphone camera is a readily available compact detection device that is suitable for on-site measurement. As bioluminescence systems do not require excitation light, they can record the signal directly without the use of the light source with a specific wavelength and emission filters. Therefore, a combination of the smartphone camera and an indicator based on bioluminescence has the potential to make a specialized inspection easier and more accessible. To demonstrate this, we tried to determine WH using LOTUS-W and a smartphone camera. The purified LOTUS-W protein was combined with commercially available bottled waters with different WH values (Table S3). The samples were placed on a microplate, and bioluminescence images were acquired by using a smartphone camera. Upon the addition of substrate, bioluminescence generated from each well was detected (Figure 4A). By separating the image color into red-green-blue, the ratios of green to blue were calculated. The built-in function of a smartphone, “auto color balance (white balance)” adjusts the gain factor of each color channel automatically. Therefore, if the ratio of the gains between blue and green color channels is changed by the function, the measured intensity ratio of the LOTUS-W is also changed, and that influences the WH determination. To avoid such an unexpected devise specific error, the function should be disabled and the color balance should be set manually. The smartphone can set the color balance between 2300K and 10000K. As a result of examining a value that was not different from the actual red-green-blue ratio, the color balance was set between 5000 and 5500K. The ratio of bioluminescent intensities increased depending on the increase of WH concentrations, and waters were successfully categorized into different levels of WH (Figure 4B). We used tap water from Osaka (Toyokawa area) in Japan, which had a hardness level of 36–41 mg/L, and it was categorized as soft water (WH < 60 mg/L), which was successfully demonstrated. This result suggested that under the same manners, LOTUS-W could be applicable to assess WH in household waters.

Thus, the portable bioluminescence indicator, LOTUS-W, can be set up on-site to measure WH in household waters. The time from sample preparations to the acquisition of the final image was estimated to be within 1–2 min. This is a practical performance time for on-site applications.

## 4. Conclusions

In this study, we constructed a noble BRET indicator LOTUS-W for the detection of WH in household waters. We have demonstrated the BRET ratio of commercially available bottled waters WH on relative quantification methods. A smartphone-based camera was introduced to detect the bioluminescence and reflect its values. This detection system offers to instantly analyze the images of WH effectively by using a digital color image analyzing system [19]. Urbanization and industrialization are rapidly expanding around the world. Many countries have been using water softening systems in their municipal drinking water [44]. It is important to verify the level of WH in groundwater or surface waters in residential areas. This can be achieved by developing a survey method by employing LOTUS-W. LOTUS-W could also be applicable to detect WH in natural water obtained from ponds, rivers, lakes, or groundwater without any pre-preparation of the sample. The sensitivity to specific ions such as Ca^2+^ or Mg^2+^ in LOTUS-W would need to be optimized by engineering the indicator domain for the practical applicability of this indicator. The combinatorial use of this indicator with different affinities for Ca^2+^ and Mg^2+^ has the possibility to compensate for the distortion caused by different affinities for Ca^2+^/Mg^2+^ in LOTUS-W, and improve the measurement accuracy. We estimated the testing cost for a single measurement is approximately 10 times higher than current commercially available kits. The higher cost compared to conventional products is an issue that will have to be solved in the future. A simple inspection method along with LOTUS-W and portable devices can be used as a potential tool in various applications of WH.

## Supplementary Materials

The following are available online at https://www.mdpi.com/1424-8220/20/11/3164/s1, Figure S1: Construction, and screening of LOTUS-W candidates, Figure S2: In vitro properties of cpV229N–HsCen3-NL variants, Figure S3: pH dependence of cpV229N_HsCen3∆C7-NL, Figure S4: Temperature dependence of cpV229N_HsCen3∆C7-NL, Figure S5: Time course variation of the LOTUS-W ratio, Table S1: Oligonucleotide sequence, Table S2. WHO/USGS-proposed water hardness scale based on CaCO_3_, Table S3. Water hardness of commercially available bottled waters, Table S4. In vitro properties of cpV229N_Cen3 variants.

## Accession Code

The nucleotide sequences of LOTUS-W deposited in DDBJ under accession number LC521885.
