# Supplementary material for: Bioluminescent Ratiometric Indicator for Analysis of Water Hardness in Household Water
Source: Sensors (Basel). 2020 Jun 2;20(11):3164. doi: 10.3390/s20113164 (PMC7308811; doi:10.3390/s20113164)
Supplement: Supplementary file 1 [file sensors-20-03164-s001.pdf]

sensors

Supporting information

Bioluminescent Ratiometric Indicator for Analysis of Water Hardness in Household Water

Md Nadim Hossain [1,2], Ryuichi Ishida [1], Mitsuru Hattori [1,2], Tomoki Matsuda [1,2] and Takeharu Nagai [1,2,*]

[1] The Institute of Scientific and Industrial Research (SANKEN), Osaka University, 8-1 Mihogaoka, Ibaraki 567-0047, Japan; nd6@sanken.osaka-u.ac.jp (M.N.H.); i.doradoradora1@gmail.com (R.I.); 8tr@sanken.osaka-u.ac.jp (M.H.); tmatsuda@sanken.osaka-u.ac.jp (T.M.)

[2] Department of Biotechnology, Graduate School of Engineering, Osaka University, 2-1 Yamadaoka, Suita 565-0871, Japan

* Correspondence: ng1@sanken.osaka-u.ac.jp

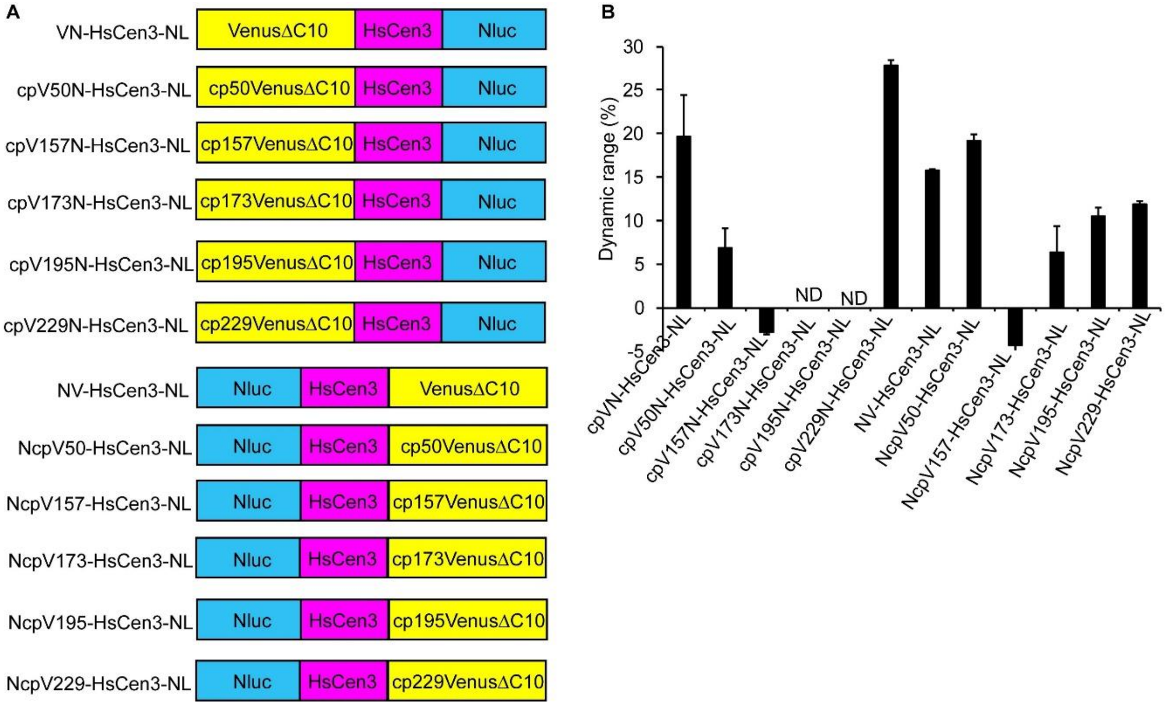

Figure S1. Construction and screening of LOTUS-W candidates (A) Domain structures of the LOTUS-W candidates. Venus or cpVenus (cp50, 157, 173, 195, or 229) was connected to the N-terminal or C-terminal of HsCen3. Nluc was connected to the other side. (B) Dynamic range for Mg^{2+} in candidates calculated in the presence and absence of 10 mM Mg^{2+} emission ratio (525 nm/455 nm). Each dynamic range is shown as a percentage (%).

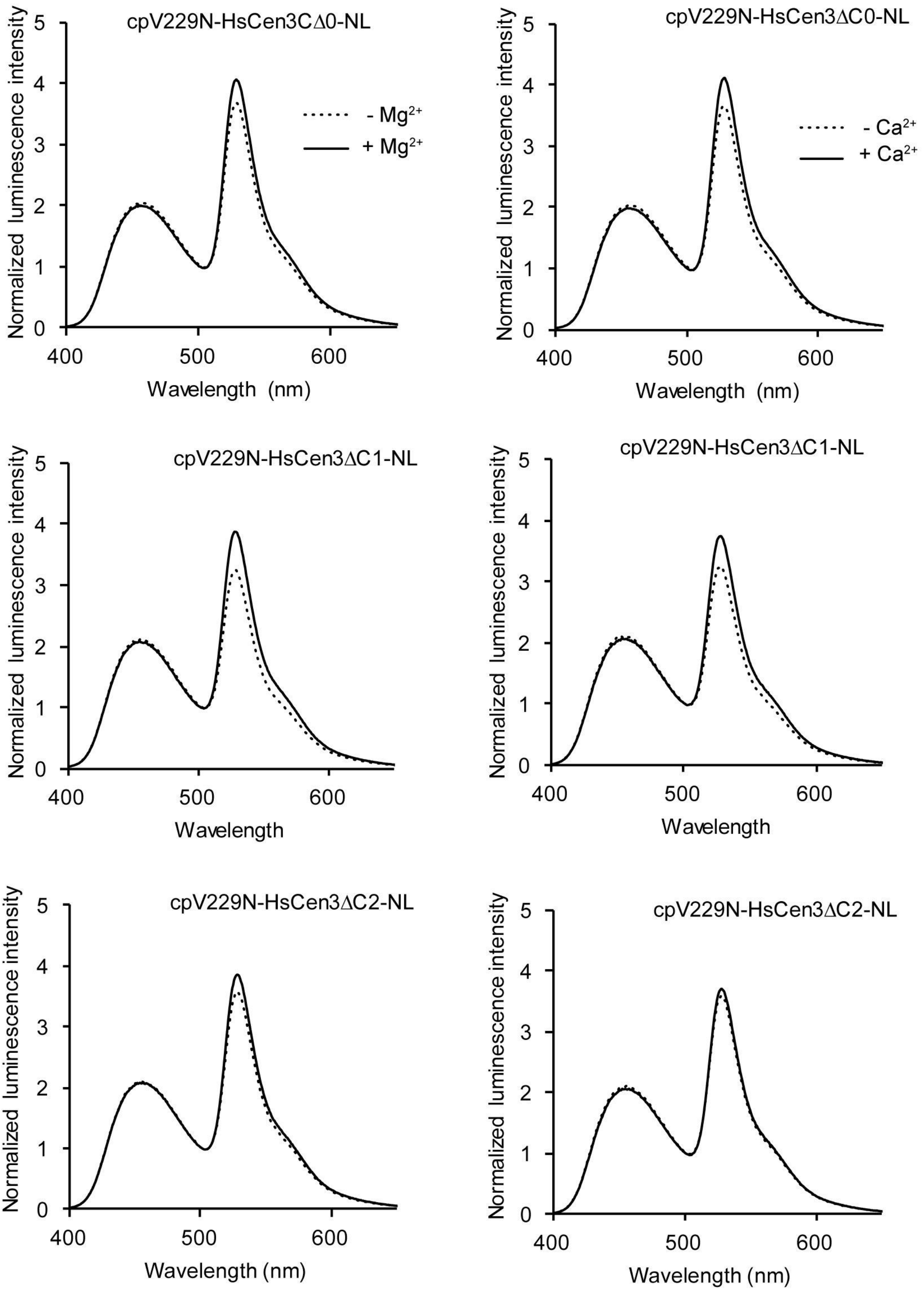
cpV229N-HsCen3CΔ0-NL
- Mg²⁺
+ Mg²⁺
Normalized luminescence intensity
Wavelength (nm)
cpV229N-HsCen3ΔC0-NL
- Ca²⁺
+ Ca²⁺
Normalized luminescence intensity
Wavelength (nm)
cpV229N-HsCen3ΔC1-NL
Normalized luminescence intensity
Wavelength
cpV229N-HsCen3ΔC1-NL
Normalized luminescence intensity
Wavelength
cpV229N-HsCen3ΔC2-NL
Normalized luminescence intensity
Wavelength (nm)
cpV229N-HsCen3ΔC2-NL
Normalized luminescence intensity
Wavelength (nm)

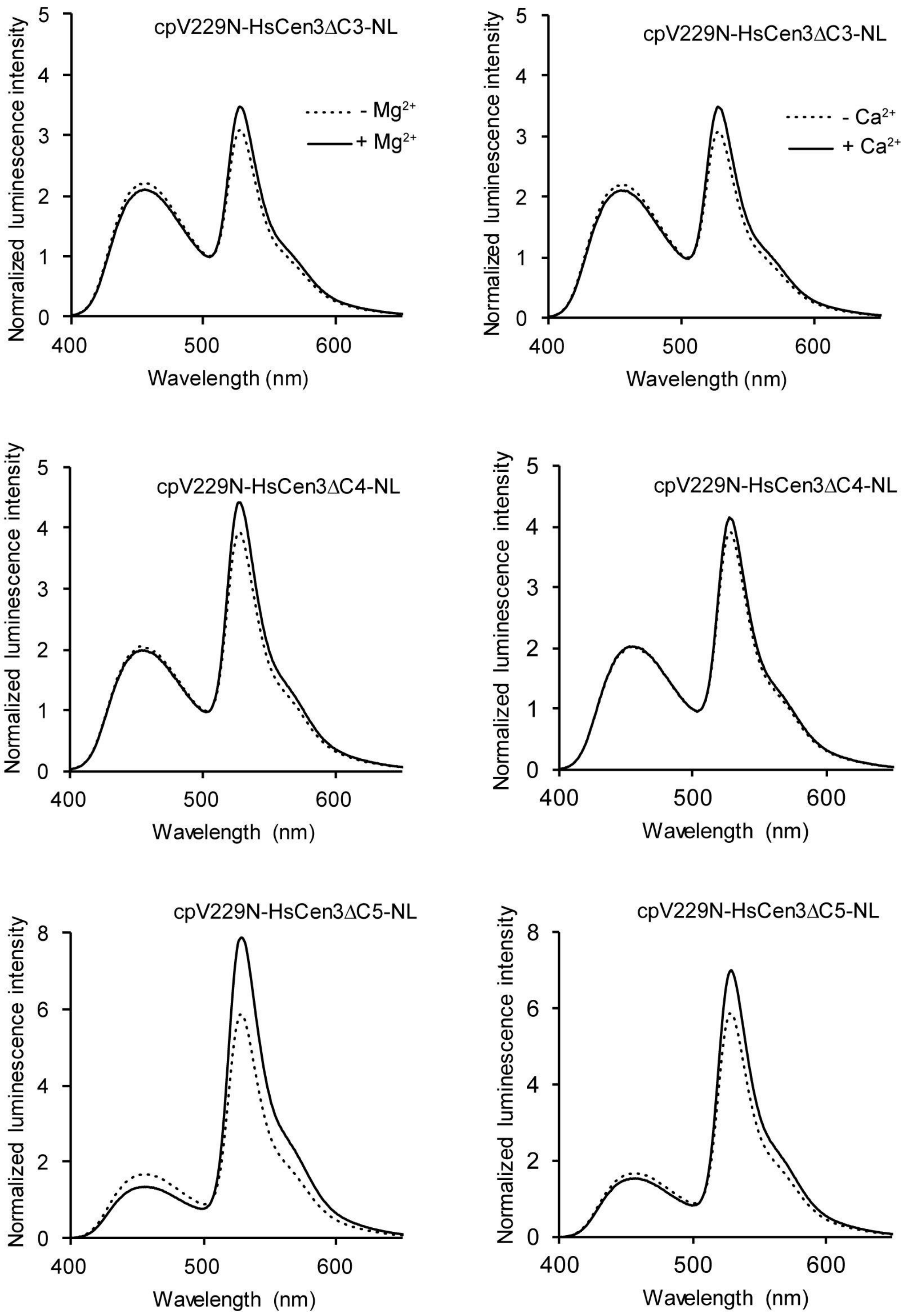
cpV229N-HsCen3ΔC3-NL
- Mg^{2+}
+ Mg^{2+}
Nomralized luminescence intensity
Wavelength (nm)
cpV229N-HsCen3ΔC3-NL
- Ca^{2+}
+ Ca^{2+}
Normalized luminescence intensity
Wavelength (nm)
cpV229N-HsCen3ΔC4-NL
Normalized luminescence intensity
Wavelength (nm)
cpV229N-HsCen3ΔC4-NL
Normalized luminescence intensity
Wavelength (nm)
cpV229N-HsCen3ΔC5-NL
Normalized luminescence intensity
Wavelength (nm)
cpV229N-HsCen3ΔC5-NL
Normalized luminescence intensity
Wavelength (nm)

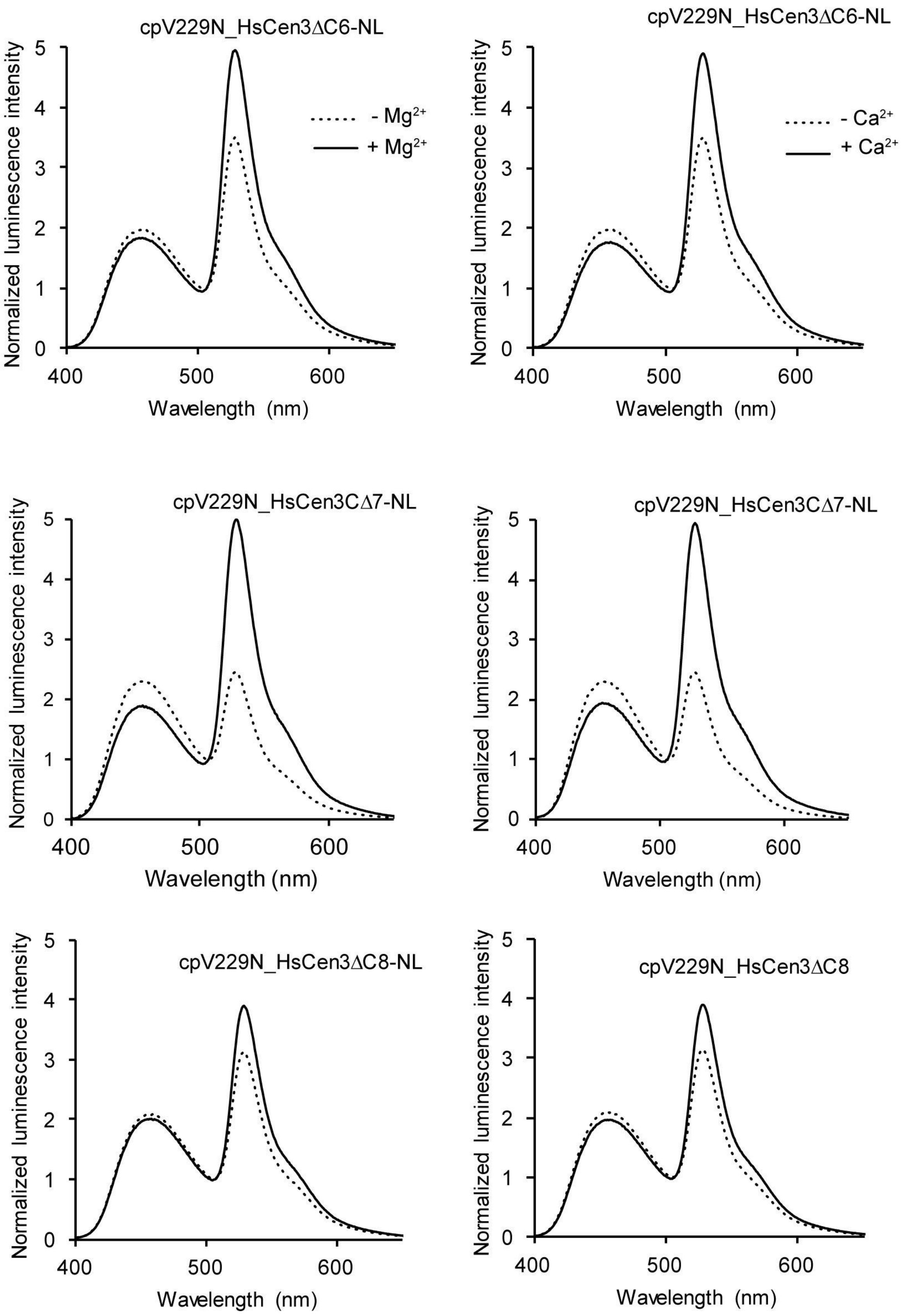
cpV229N_HsCen3ΔC6-NL
- Mg2+
+ Mg2+
cpV229N_HsCen3ΔC6-NL
- Ca2+
+ Ca2+
cpV229N_HsCen3CΔ7-NL
cpV229N_HsCen3CΔ7-NL
cpV229N_HsCen3ΔC8-NL
cpV229N_HsCen3ΔC8
Normalized luminescence intensity
Wavelength (nm)
0
1
2
3
4
5
400
500
600

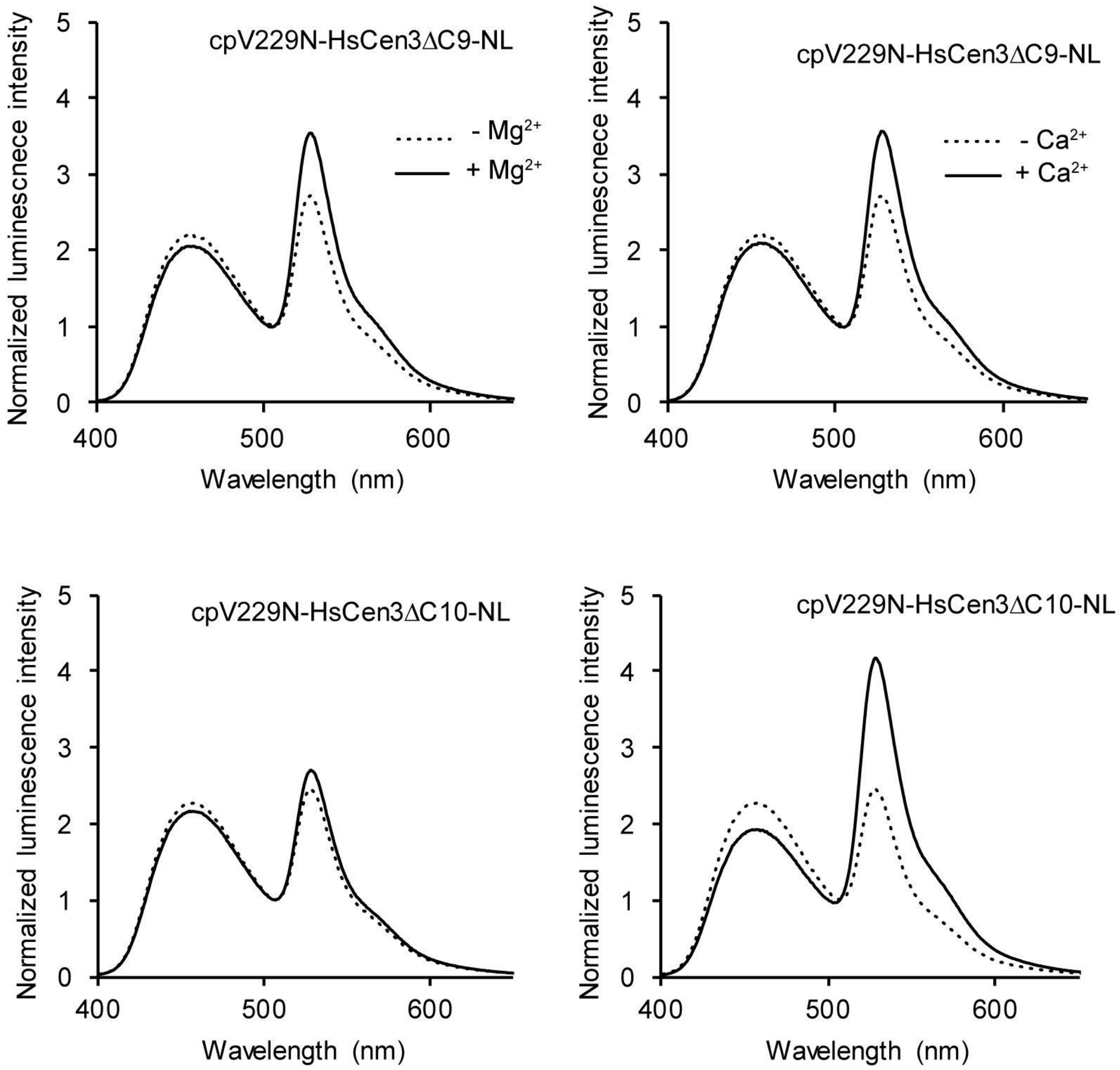

Figure S2. *In vitro* properties of cpV229N–HsCen3-NL variants. Bioluminescence spectra of cpV229N–HCen3-N3 variants are shown in the presence (solid line) or absence (dotted line) of 10 mM Mg^{2+} or Ca^{2+}.

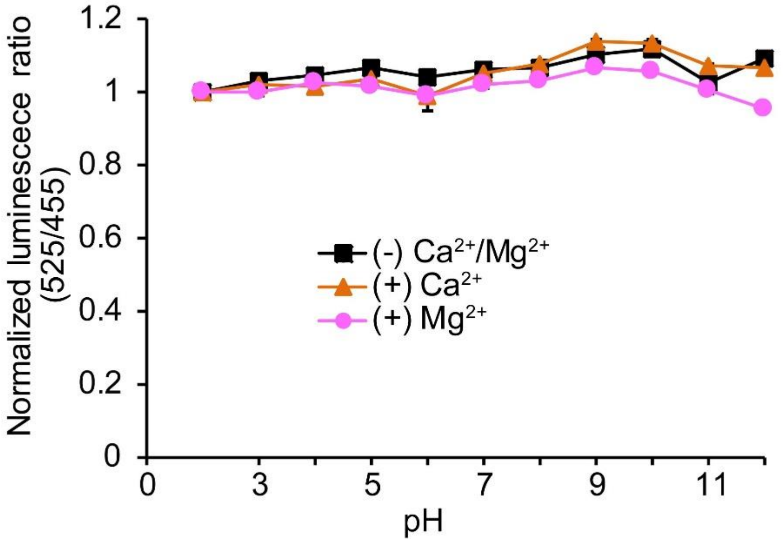

Figure S3. pH dependence of cpV229N_HsCen3ΔC7-NL. Bioluminescence intensity ratios of cpV229N_HsCen3ΔC7-NL were confirmed at different pH values. These counts were measured in the presence and absence of 10 mM Ca^{2+} or 10 mM Mg^{2+}. All measurements were performed three times (n = 3).

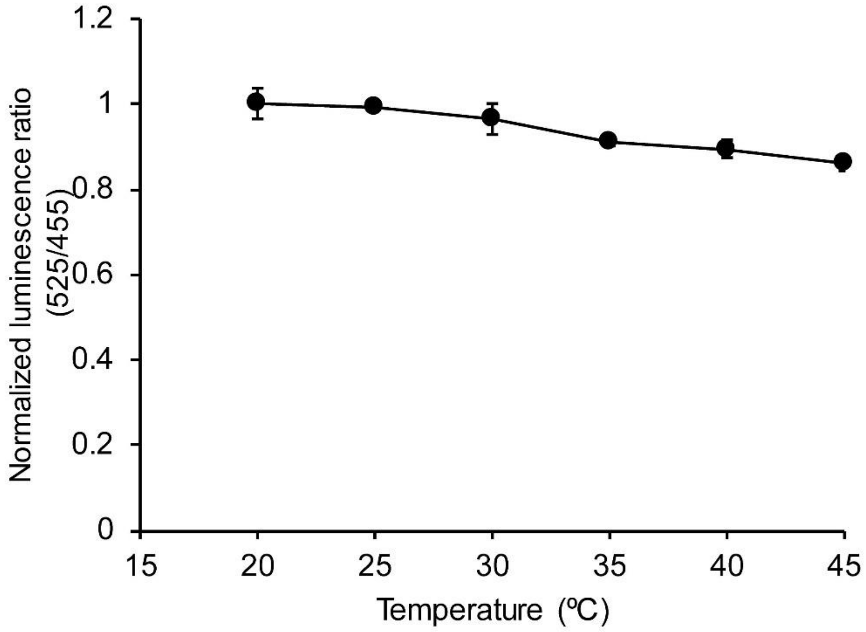

Figure S4. Temperature dependence of cpV229N_HsCen3ΔC7-NL. Bioluminescence intensity ratio at different temperatures.

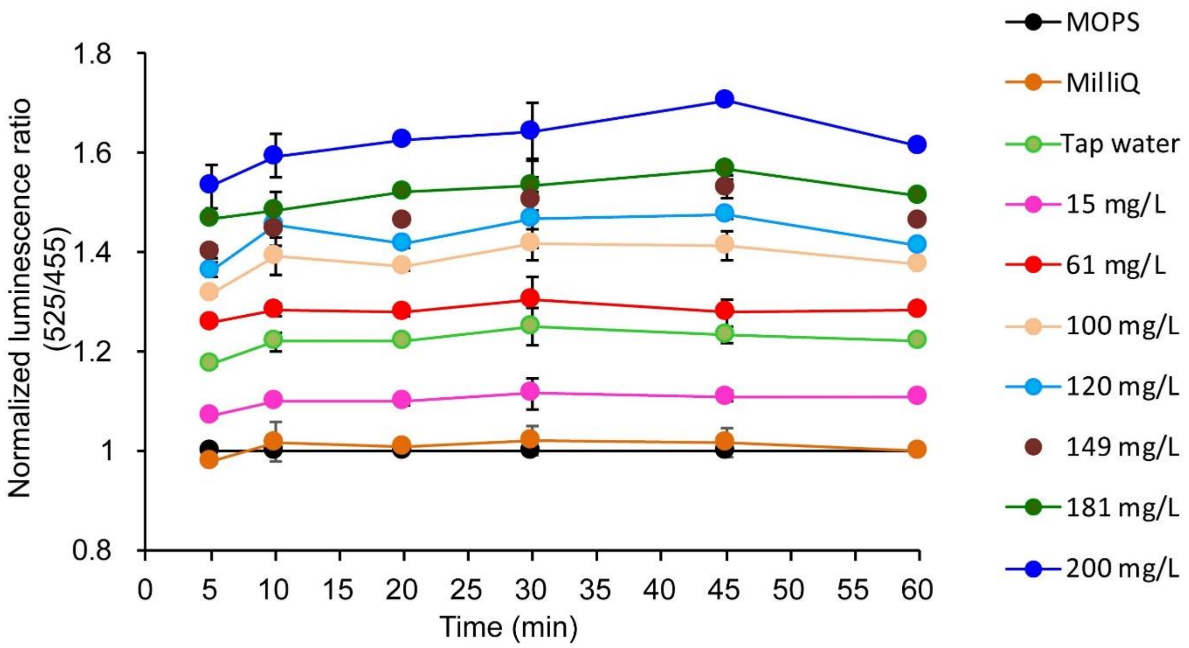

Figure S5. Time course variation of the LOTUS-W ratio. Bioluminescence intensities of LOTUS-W were measured at each time point with different water hardness ($CaCO_3$) conditions and the ratio was calculated (525 nm/455 nm). All ratios were normalized to 10 mM MOPS without added ions.

Table S1. Oligonucleotide sequence.

Name	Sequence (5'--3')
F-BH1I-Venus_1	GCGGATCCGATG GTGAGCAAGGGCGAG
F-BH1-M-ven_50	AAGGATCCGATGACCGGCAAGCTGCCC
F-BH1-M-ven_157	AAGGATCCGAGCAGAAGAACGGCATC
F-BH1-M-ven_173	AAGGATCCGATGGACGGCGGCGTGC
F-BH1-M-ven_195	TTGGATCCGATGCTGCCCGACAACCAC
F-BH1-M-ven_229	AAGGATCCGATGATCACTCTCGGCATG
R-ven_49-SphI	ATTGCATGCGGGTGCAGATCAGCTTCAGGG
R-ven_156-SphI	GACGCATGCGCTTGTCGGCGGTGATATAGA
R-ven_172-SphI	ATTGCATGCGCTCGATGTTGTGGCGGATCT
R-ven_194-SphI	ATTGCATGCGCAGCACGGGGCCGTCGCCGA
R-ven_228-SphI	TATGCATGCGCCCGGCGGCGGTCACGAACT
F-SacI-gfp_1	TTTGAGCTCATGGTGAGCAAGGGCGAGGA
F-SacI-M-ven_50	AATGAGCTCATGACCGGCAAGCTGCCCGTGCCC
F-SacI-M-ven_157	AATGAGCTCATGCAGAAGAACGGCATCAAGGCCAAC
F-SacI-M-ven_173	AATGAGCTCATGGACGGCGGCGTGCAGCTCGCC
F-SacI-M-ven_195	AATGAGCTCATGCTGCCCGACAACCACTACCTGAGC
F-SacI-M-ven_229	TTTGAGCTCATGATCACTCTCGGCATGGA
R-ven_49-x-EcoRI	ATTGAATTCTTAGGTGCAGATCAGCTTCAG
R-ven_156-x-EcoRI	ATTGAATTCTTACTTGTCGGCGGTGATATA
R_ven_172-x-EcoRI	ATTGAATTCTTACGATGTTGTGGCGGATCT
R-ven_195-x-EcoR1	ATTGAATTCTTACAGCACGGGGCCGTCGCC
R-ven_229-x-EcoR1	ATTGAATTCTTACCCGGCGGCGGTCACGAA
R-cp229Venus-Sph1	ATAGCATGCCCCGGCGGCGGTCACGAA
R-Nluc-171-X-ER1	TATGAATTCTTACGCCAGAATGCGTTCGC
R-Nluc-171-g-SphI	ATTGCATGCGCGCCAGAATGCGTTCGCACA
R-BH1-g-Nluc	AATGGATCCGATGGTCTTCACACTCGAAG
F-SacI_Nanoluc	ATCGAGCTCGATGGTCTTCACACTCGA
R_SacI_hsCen3-ΔC1	TGGGAGCTCATCTCTTTCCAATATCCAGTCTG
R_SacI_hsCen3-ΔC2	TGGGAGCTCTCTTTCCAATATCCAGTCTGTC
R_SacI_hsCen3-ΔC3	TGGGAGCTCTTCCAATATCCAGTCTGTCAC
R_SacI_hsCen3-ΔC4	TGGGAGCTCCAATATCCAGTCTGTCACAACT
R_SacI_hsCen3-ΔC5	TGGGAGCTCTATCCAGTCTGTCACAACTTC
R_SacI_hsCen3-ΔC6	TGGGAGCTCCCAGTCTGTCACAACTTCATT
R_SacI_hsCen3-ΔC7	TGGGAGCTCCCAGTCTGTCACAACTTCATT
R_SacI_hsCen3-ΔC8	TGGGAGCTCTGTCACAACTTCATTAAAATCTTCA
R_SacI_hsCen3-ΔC9	TGGGAGCTCCACAACTTCATTAAAATCTTCAAAGG
R_SacI_hsCen3-ΔC10	TGGGAGCTCAACTTCATTAAAATCTTCAAAGGTGA

Table S2. WHO/USGS-proposed water hardness scale based on $CaCO_3$.

Scale of WH (mg/L)	Classification
0–60	Soft
61–120	Moderately hard
121–180	Hard
181<	Very hard

Table S3. Water hardness of commercially available mineral water.

Product name	Scale of WH (mg/L)	Classification	Mg^{2+} (mg/L)	Ca^{2+} (mg/L)	Other minerals	pH	Company name, Country
Natural Mineral Water	10	Soft	0.5	3	—	6.4	AEON CO., LTD., Japan
Okudaisan no Tennensui	20	Soft	1~3	2~7	Na^+, K^+	7	Suntory Holdings Limited, Japan
Minami-Alps no Tennensui	30	Soft	1~3	6~15	Na^+, K^+	7	Suntory Holdings Limited, Japan
CRYSTAL Geyser	38	Soft	3.4	6.4	Na^+, K^+	—	Crystal Geyser Water Company, USA
Irohasu	40	Soft	5.5	7.1	Na^+, K^+	7	The Coca-Cola Company, Japan
Volvic	60	Soft	8	10.15	Na^+, K^+	7	Danone S.A., France
Ogeu	172	Hard	10.2	40.6	K^+		Coron Vert CO., LTD., France
Beaupre	240	Very hard	20.4	50.8	—	—	CMA CGM, France
Evian	304	Very hard	20.6	80	Na^+	7.2	Danone S.A., France
Vittle	315	Very hard	20	90.4	—	—	Nestlé S.A., France
Perlage	389	Very hard	20.4	110.7	K^+	5.3	Nałęczów Zdrój Sp. z o.o, Poland
S.Pellegrino	610	Very hard	40.9	160.4	So_4^{2-},HCO_3^-, Cl^-	—	Sanpellegrino S.p.A, Italy
Gerolsteiner	1310	Very hard	100	360	Na^+, K^+	6.4	GmbH & Co. KG, Germany
Contrex	1468	Very hard	70.45	460.8	K^+	—	Nestlé S.A., France

Table S4. *in vitro* properties of cpV229N_HsCen3-NL variants.

Variants name	R_{min} for Mg^{2+}/Ca^{2+}	R_{max} for Mg^{2+}	R_{max} for Ca^{2+}	Dynamic range for Mg^{2+} (%)	Dynamic range for Ca^{2+} (%)
cpV229N_Cen3-NL	1.8	2	2	13	15
cpV229N_Cen3ΔC1-NL	1.5	1.9	1.8	23	18
cpV229N_Cen3ΔC2-NL	1.7	1.9	1.8	9	6
cpV229N_Cen3ΔC3-NL	1.3	1.6	1.7	18	18
cpV229N_Cen3ΔC4-NL	1.9	2.2	2	15	5
cpV229N_Cen3ΔC5-NL	3.4	5.8	4.5	66	30
cpV229N_Cen3ΔC6-NL	1.7	2.7	2.8	52	56
cpV229N_Cen3ΔC7-NL	1	2.6	2.6	145	140
cpV299N_Cen3ΔC8-NL	1.5	1.9	2	29	31
cpV229N_Cen3ΔC9-NL	1.2	1.7	1.7	40	38
cpV229N_Cen3ΔC10-NL	1	1.2	2.2	16	101
